# Cryo-EM structure of a SARS-CoV-2 omicron spike protein ectodomain

**DOI:** 10.1038/s41467-022-28882-9

**Published:** 2022-03-03

**Authors:** Gang Ye, Bin Liu, Fang Li

**Affiliations:** 1 Department of Veterinary and Biomedical Sciences, University of Minnesota, Saint Paul, MN USA; 2 Center for Coronavirus Research, University of Minnesota, Saint Paul, MN USA; 3 Hormel Institute, University of Minnesota, Austin, MN USA

**Keywords:** SARS-CoV-2, Cryoelectron microscopy

## Abstract

The omicron variant of SARS-CoV-2 has been spreading rapidly across the globe. The virus-surface spike protein plays a critical role in the cell entry and immune evasion of SARS-CoV-2. Here we determined the 3.0 Å cryo-EM structure of the omicron spike protein ectodomain. In contrast to the original strain of SARS-CoV-2 where the receptor-binding domain (RBD) of the spike protein takes a mixture of open (“standing up”) and closed (“lying down”) conformations, the omicron spike molecules are predominantly in the open conformation, with one upright RBD ready for receptor binding. The open conformation of the omicron spike is stabilized by enhanced inter-domain and inter-subunit packing, which involves new mutations in the omicron strain. Moreover, the omicron spike has undergone extensive mutations in RBD regions where known neutralizing antibodies target, allowing the omicron variant to escape immune surveillance aimed at the original viral strain. The stable open conformation of the omicron spike sheds light on the cell entry and immune evasion mechanisms of the omicron variant.

## Abstract (web summary)

This study determined the structure of the spike protein of the SARS-CoV-2 omicron variant, revealing a predominantly open conformation of the molecule that may help omicron infect cells more efficiently than do previous variants.

## Introduction

**Fig. 1:**
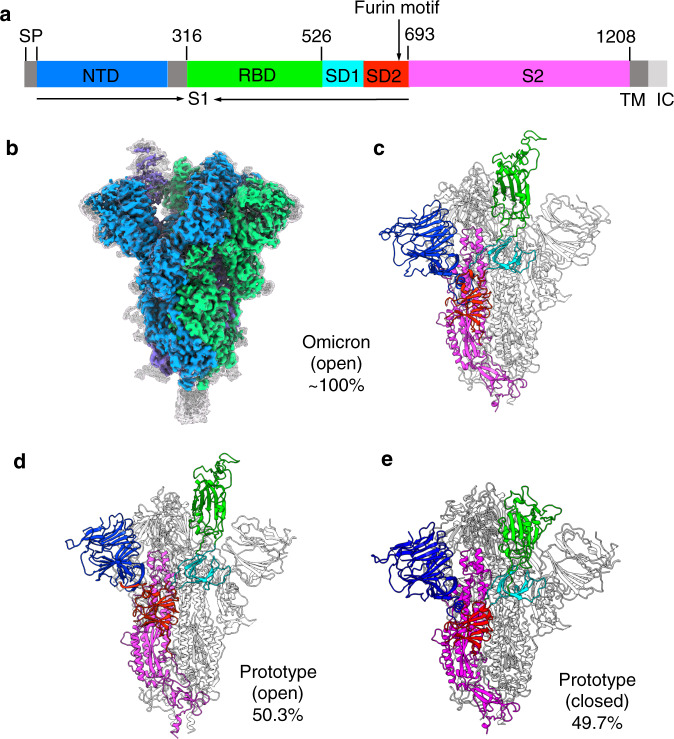
Overall structures of the omicron and prototypic spike proteins. **a** The schematic representation of full-length omicron spike. SP signal peptide, NTD N-terminal domain, RBD receptor-binding domain, SD1 subdomain 1, SD2 subdomain 2, TM transmembrane anchor, IC intracellular tail. The Furin cleavage site is indicated by the arrow. **b** Superimposition of sharpened and unsharpened cryo-EM maps for the omicron spike ectodomain. The unsharpened map is shown in the gray mesh. The sharpened map is shown in three different colors, one for each of the spike subunits. **c** Structural model of the omicron spike ectodomain in the open conformation. **d** Structural model of the prototypic spike ectodomain in the open conformation. **e** Structural model of the prototypic spike ectodomain in the closed conformation. For the structural models, two of the three spike subunits are colored in gray and the other spike subunit is colored by its domains: NTD in blue, RBD in green, SD1 in cyan, SD2 in red, and S2 in magenta. The population distributions for each spike ectodomain based on its RBD conformation were presented as percentages.

The newly emerged SARS-CoV-2 omicron variant poses new challenges to global health and economy^1^. Compared to earlier strains, the omicron strain causes milder symptoms, has shorter incubation time and shorter recovery time, infects vaccinated people more easily and spreads more widely^2–4^. As SARS-CoV-2 spreads, mutations mainly occur in its envelope-anchored spike protein^5,6^. The spike mediates SARS-CoV-2 entry into human cells, induces humans’ neutralizing immune responses, and is the basis of current COVID-19 mRNA vaccines^7,8^. The spike on mature virions is a clove-shaped homotrimer (i.e., its pre-fusion structure), with three receptor-binding S1 subunits sitting on top of a trimeric membrane-fusion S2 stalk (Fig. 1a)^9,10^. Each S1 subunit contains a receptor-binding domain (RBD) and an N-terminal domain (NTD)^7^. During cell entry, the RBD binds to its host receptor, angiotensin-converting enzyme 2 (ACE2), for viral attachment^11–14^. Moreover, a short motif at the S1/S2 boundary is specifically cleaved by a human protease furin^15^. Both ACE2 binding and furin cleavage trigger S1 to dissociate^16,17^. Then S2 undergoes a dramatic structural change to fuse viral and host membranes (i.e., reaching its post-fusion structure)^18^. Because of its important roles, the spike holds the key to understanding the cell entry and immune evasion of the omicron strain.

The conformation of the RBD in the trimeric SARS-CoV-2 spike protein is an important molecular determinant of the COVID-19 pandemic^15^. The RBD is present in two different conformations: standing up for receptor binding and lying down for immune evasion. Correspondingly, the spike molecule is in the open and closed conformation, respectively. Compared to the closed spikes, the open spikes mediate receptor binding and cell entry more effectively, but also expose the RBD to the immune system. Numerous structural studies have characterized the RBD conformations of the spike proteins from different SARS-CoV-2 variants (Supplementary Table 1)^10,18–27^. Despite some inconsistencies due to differences in experimental procedures, a prevailing finding is that a D614G mutation near the S1/S2 boundary and some other mutations in a recent delta strain allowed more spike molecules to open up, relative to the original Wuhan strain^10,18–27^. The omicron spike contains many mutations^5,6^, but their impact on the conformation of the spike protein or its immune evasion is unclear. In this study, we determined the cryo-EM structure of the omicron spike ectodomain at 3.0 Å resolution. We also determined the cryo-EM structure of the prototypic spike ectodomain (Wuhan strain plus the D614G mutation). We compared the conformations of these two spike ectodomains and discussed how the conformation of the omicron spike affects the cell entry and immune evasion of the omicron variant and the future trajectory of the COVID-19 pandemic.

## Results

To determine the structure of the omicron spike, we expressed its ectodomain in mammalian cells and purified it to high homogeneity (Supplementary Fig. 1a). To stabilize the spike ectodomain in its pre-fusion structure, proline mutations were introduced to the S2 subunit^28^. To better represent its conformation on virion surfaces, the furin motif at the S1/S2 boundary was preserved. As a control, we also prepared a prototypic spike ectodomain (Wuhan strain plus D614G mutation) in a similar way. Sodium dodecyl sulfate polyacrylamide gel electrophoresis of the two purified recombinant spikes showed that almost all of the omicron spike molecules and about half of the prototypic spike molecules had been cleaved by furin, suggesting that the furin motif in the recombinant omicron spike ectodomains is more efficient than that in the prototypic spike ectodomain. It remains to be seen whether the above observation on the furin cleavage can be extended to live omicron virions. Negative-stain EM revealed that both of the recombinant omicron and prototypic spike ectodomains were well structured (Supplementary Fig. 1b).

We collected cryo-EM data for both of the spike ectodomains (Supplementary Table 2). The global 3D classification was performed to determine the conformations of the RBDs in each of the spikes (Supplementary Figs. 2–5). For the omicron spike, ~100% of the spike particles were in the open conformation, with one RBD in the standing up position and two RBDs in the lying down position; a 3.0 Å map was calculated for the omicron open spike, clearly defining the density of the standing up RBD (Fig. 1b). Further focused 3D classification was performed on those particles in the open conformation, also displaying that no omicron spike particles were in the closed conformation (Supplementary Fig. 2). This result was further confirmed by 3D variability (Supplementary Movies 1-4). As a comparison, for the prototypic spike, 50.3% of the spike particles were in the one RBD-up open conformation based on a 3.2 Å map, and 49.7% of the spike particles were in the closed conformation based on a 3.0 Å map (Supplementary Fig. 3). This result was further confirmed by 3D variability analysis (Supplementary Movies 5–10). Structural models for the omicron open spike, prototypic open spike, and prototypic closed spike were built and refined based on these cryo-EM maps (Fig. 1c; Fig. 1d; Fig. 1e; Supplementary Figs. 2–6). Therefore, compared to the prototypic spike, more omicron spike molecules are in the open conformation.

**Fig. 2:**
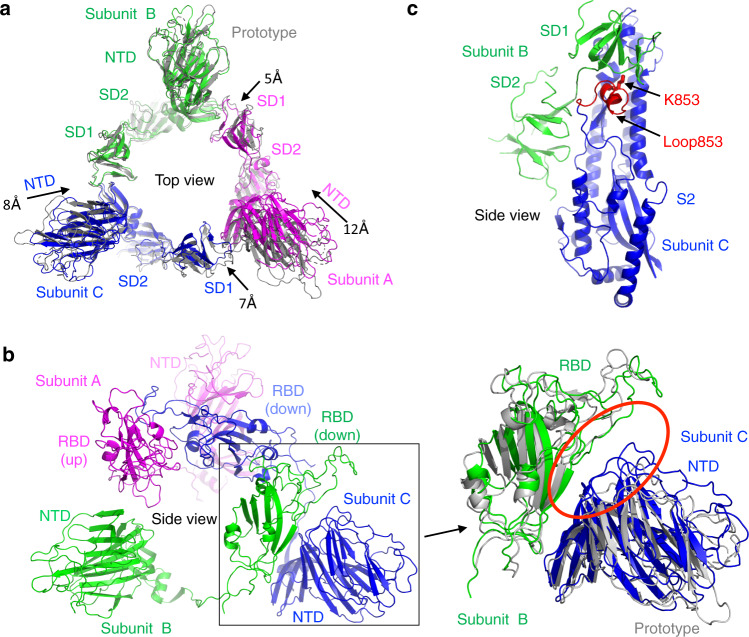
Structural comparisons of omicron open spike and prototypic open spike. **a** Structural superimposition of the S1 subunits (without the RBDs) of omicron open spike and prototypic open spike from the top view. Prototypic spike is colored in gray. Omicron spike is colored by different subunits: subunit A contains the up-RBD and is colored in magenta, and subunits B and C contain the down-RBD and are colored in green and blue, respectively. The arrows indicate the domain shifting of the omicron spike relative to the prototypic spike. **b** Structural superimposition of the S1 subunit (without SD1 or SD2) of omicron open spike and prototypic open spike from the side view. The structures are colored in the same way as in panel (**a**). The interface between the NTD from subunit C and the RBD from subunit B is highlighted in the red circle. Because of domain shifting as shown in panel (**a**), the RBD/NTD interfaces are larger in the omicron open spike than in the prototypic open spike. **c** The S1/S2 structural interface in the omicron open spike from the side view. The structure is colored in the same way as in panel (**a**). Loop 853 (an S2 loop spanning residues 831–854) and Lys853 (which differs from the corresponding residue in the prototypic spike) are both colored in red. Loop 853 is partially disordered in the prototypic spike, but becomes ordered and mediates S1/S2 interactions in the omicron spike.

To understand the structural basis for the omicron open spike, we analyzed the structural packing of the molecule. Like other β-genus coronaviruses, SARS-CoV-2 S1 takes a cross-subunit packing mode where the RBD of one subunit packs against the NTD of another subunit^29^. Due to its mobility, the standing-up RBD has relatively weak densities. We, therefore, calculated the buried interface between each of the two lying-down RBDs and its neighboring NTD and between each of the three S1 subunits and its neighboring S2 subunit (Supplementary Table 3). Compared to the prototypic open spike, the omicron open spike had significantly increased buried interface between the lying-down RBDs and their neighboring NTD and between the S1 subunits and their neighboring S2. Hence, the omicron open spike is packed significantly more tightly than the prototypic open spike. Structural overlay of the omicron open spike and prototypic open spike revealed that compared to the prototypic open spike, the NTD and the SD1 of the omicron open spike both shifted towards the neighboring S1 subunit (Fig. 2a). As a result of these domain movements, each of the lying-down RBDs packs more tightly with its neighboring NTD in the omicron open spike (Fig. 2b). Moreover, a partially disordered loop (i.e., loop 853 as described below) in S2 of the prototypic open spike becomes structurally ordered in the omicron open spike, mediating contacts between S1 and S2 (Fig. 2c). Overall, the omicron open spike packs more tightly than the prototypic open spike.

**Fig. 3:**
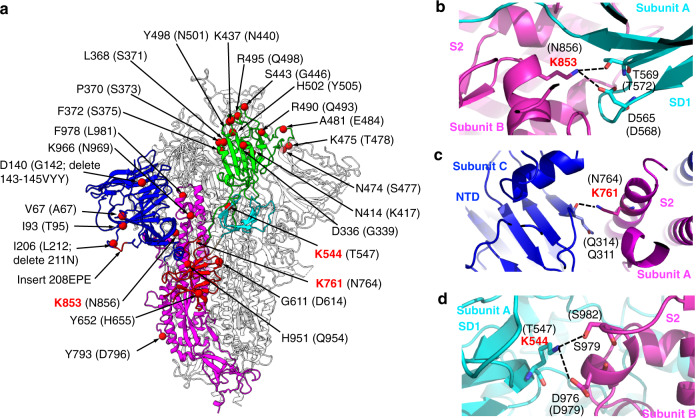
Mutations in the omicron spike introduce new inter-domain and inter-subunit interactions that may stabilize its open conformation. **a** Structural distribution of all of the mutations in the omicron spike ectodomain. The omicron open spike is colored in the same way as Fig. 1c. All of the mutations are shown in the red sphere and labeled with their residue numbers as in the omicron spike (the corresponding residue numbers in the prototypic spike are shown in parentheses). Three residues that introduce new inter-domain and inter-subunit interactions are highlighted in red. **b**–**d** New inter-domain and inter-subunit interactions in the omicron open spike are mediated by three new mutations. The omicron open spike is colored in the same way as Fig. 2a. The dashed lines represent salt bridges and hydrogen bonds.

To understand the molecular force driving the above domain shifting, we analyzed how the mutations affected the structural packing of the omicron open spike (Fig. 3a). A number of mutations in the omicron spike are distributed at the inter-domain and inter-subunit surfaces. Particularly, Asn856 in the prototypic spike becomes Lys853 in the omicron spike, which forms a hydrogen bond and a salt bridge with Thr569 and Asp565 from SD1, respectively (Fig. 3b). Because Lys853 is located on the loop at the S1/S2 interface (i.e., loop 853), this mutation may help loop 853 become more ordered and S1/S2 packing become tighter. Moreover, Asn764 and Thr547 in the prototypic spike become Lys761 and Lys544 in the omicron spike, respectively: Lys761 is located in S2 and forms a hydrogen bond with the main chain of Gln311 from the NTD (Fig. 3c), whereas Lys544 is located in SD1 and forms a hydrogen bond and a salt bridge with Ser979 and Asp976 from S2, respectively (Fig. 3d). These mutations may also enhance S1/S2 packing. Overall, these mutations in the omicron spike introduce new inter-domain and inter-subunit interactions, which may stabilize the open conformation of the omicron spike.

**Fig. 4:**
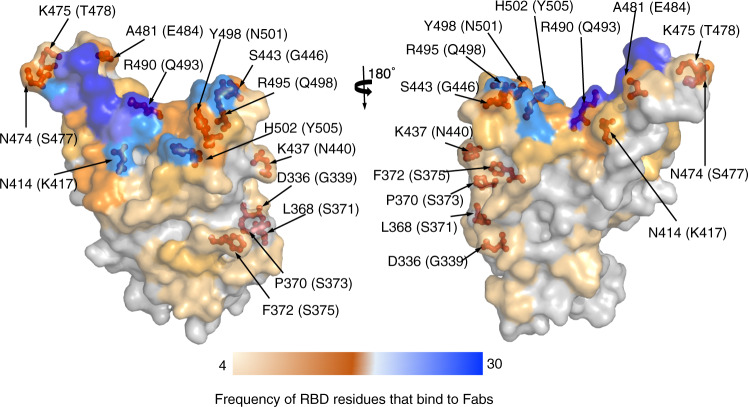
Mutations in the omicron spike RBD that potentially allow the omicron variant to escape from known neutralizing antibodies. Forty-nine PDBs of neutralizing antibody/RBD complexes were analyzed using PDBePISA (https://www.ebi.ac.uk/pdbe/pisa/). RBD residues are colored based on their frequency in interacting with known neutralizing antibodies: from light orange (low frequency) to blue (high frequency). RBD residues that have undergone mutations in the omicron RBD are shown as red sticks. Structural data for neutralizing antibody/RBD complexes were obtained from the PDB: 6wpt, 6xc2, 6xc4, 6xcm, 6xdg, 6xkp, 6xkq, 6yor, 7a5r, 7akd, 7b3o, 7bwj, 7byr, 7c01, 7cac, 7cdi, 7cdj, 7ch4, 7ch5, 7chb, 7chh, 7cho, 7chp, 7chs, 7cjf, 7eam, 7ean, 7jx3, 7k8m, 7k8v, 7k8w, 7k8x, 7k43, 7lrs, 7m6d, 7m6f, 7m6g, 7m6h, 7m7w, 7m42, 7mkl, 7mlz, 7n4i, 7n4j, 7n4l, 7n4m, 7r6w, 7r7n, 7sn2. Fab: antigen-binding fragment.

To understand the immune evasion of the omicron spike, we analyzed the distribution of the mutations in the omicron spike RBD relative to the binding sites of known neutralizing antibodies. To this end, we mapped all of the RBD residues that directly interact with 49 known neutralizing antibodies (Fig. 4). We also calculated the frequency of each of the RBD residues involved in interacting with these neutralizing antibodies (Fig. 4). Numerous mutations in the omicron spike overlap with the regions that interact with neutralizing antibodies with high frequency (Fig. 4). Therefore, many of the mutations that occurred in the omicron RBD allow the virus to escape from these known neutralizing antibodies. It remains to be seen how these immune escape mutations in the omicron RBD affects its binding affinity for human ACE2.

## Discussion

The emergence of the SARS-CoV-2 omicron variant has added a new twist to the COVID-19 pandemic^1^. Compared to the previous viral strains, the omicron variant manifests several unique clinical features such as milder symptoms, shorter incubation time and shorter recovery time, and more efficient infection of vaccinated people^2–4^. These features allow the omicron strain to spread fast in human populations, replacing the previous strains in many parts of the world. The spike protein plays the most critical role in the cell entry and immune evasion of SARS-CoV-2 and has been the hotspot for viral mutations^5–7^. Here we determined the cryo-EM structures of the omicron spike and a prototypic spike. Whereas the prototypic spike molecules are present in either open (50.3%) or closed conformation (49.7%), the omicron spike molecules are predominantly in the open conformation with one RBD in the standing up position. Compared to the prototypic spike, the omicron spike is packed more tightly with enhanced inter-domain and inter-subunit interactions. Numerous mutations have occurred at the inter-domain and inter-subunit surfaces in the omicron spike, resulting in the tight packing of the omicron spike. As a result, the omicron spike has a stable open conformation, in contrast to the loose packing and mixed conformations of the prototypic spike.

Our findings on the RBD conformations are in agreement with two recent studies^30,31^. Using cryo-EM, both those studies found that the recombinant omicron spike ectodomain molecules are predominantly in the open conformation^30,31^. A third study reached a different conclusion^32^, which could be due to differences in sample preparation procedures. The strength of our findings is supported by the direct comparison of the recombinant omicron spike and prototypic spike under the same conditions, minimizing the possibility that different experimental conditions have led to different population statistics of the omicron spike molecules. Additional evidence of the RBD conformations will come from future studies on the structure of full-length virion-surface-anchored omicron spike molecules. These studies will need to overcome the difficulty of achieving high-resolution images with virion structures.

Owing to extensive mutations in the RBD, the omicron variant has so far evaded immune responses established from previous infections or vaccinations. These mutations render known neutralizing antibodies ineffective, explaining a large number of breakthrough cases in vaccinated individuals. However, the stable open conformation of the omicron spike makes it a new target for vaccines and therapeutics. The stable open spike is a viral strategy for more efficient receptor binding and viral entry. Yet by exposing the RBD to the immune system, the stable open spike makes the virus more vulnerable to neutralizing immune responses. Compared to the hidden RBD in ~50% of the prototypic spike molecules, the exposed RBD in ~100% of the omicron spike molecules may allow the immune system to respond relatively quickly and effectively to viral infection, consistent with the shorter incubation time and shorter recovery time in omicron-infected patients. Thus, although the omicron strain has evaded neutralizing immune responses aimed at previous viral strains, vaccines and neutralizing antibodies specifically designed to target the omicron RBD could help bring this variant under control. Future functional and clinical studies are needed to verify the implications of the stable open omicron spike.

## Methods

### Plasmids

The gene encoding SARS-CoV-2 spike (original Wuhan strain; GenBank accession number QHD43416.1) was synthesized (GenScript Biotech). Mutations were introduced by site-directed mutagenesis to generate the prototypic SARS-CoV-2 spike gene (encoding the spike from Wuhan strain plus D614G mutation) and omicron spike gene (B.1.1.529, GISAID: EPI_ISL_6647960). The gene encoding omicron spike ectodomain (residues 14–1208) and the gene encoding prototypic spike ectodomain (residues 14–1211) were cloned into the pLenti-transfer vector (Addgene) with an N-terminal signal peptide sequence and a C-terminal foldon trimerization tag sequence followed by a His-tag sequence. To stabilize the spikes in their pre-fusion structure, proline mutations were introduced to their S2 subunit region as recommended^9,28^.

#### Protein expression and purification

Plasmids encoding the spike ectodomains were transfected into 500 ml 293F cells (Thermo Scientific) at a density of 1.0 × 10^6^ using Polyethylenimine (PEI). After 60 h, the proteins were harvested from the supernatants of cell culture medium, purified on Ni-NTA column, and purified further on Superose 6 increase gel filtration column (Cytiva).

### Negative stain electron microscopy

The spike protein samples were directly analyzed using a Tecnai Spirit Biotwin TEM (FEI). Copper grids with 300 mesh and a thin layer of continuous carbon floated on top (EM Sciences) were glow-discharged at 15 mA for 60 s. The spike protein samples were diluted to 80 nM and applied 3 µL to the grids for 30 s. Uranyl formate (80 µL 1%, wt/vol) was then used to stain the sample on the grid for 1 min. The specimen was finally gently blotted from the side with filter paper and air-dried before imaging. Images were recorded on a Gatan 4 K × 4 K CCD camera with a defocus of −2 μm at a nominal magnification of 68,000× or 98,000×.

### Cryo-EM grid preparation and data acquisition

Purified recombinant omicron spike and prototypic spike (4 µl at ~3.3 µM and ~10 µM, respectively), supplemented with 8 mM CHAPSO immediately before grid preparation, were applied to freshly glow-discharged Quantifoil R1.2/1.3 300-mesh copper grids (EM Sciences). Grids were blotted for 4 s at 22 °C under 100% chamber humidity and plunge-frozen in liquid ethane using a Vitrobot Mark IV (FEI). Cryo-EM data were collected using EPU version 2.5 (ThermoFisher Scientific) on a Titan Krios electron microscope (ThermoFisher Scientific) equipped with a Falcon III direct electron detector (ThermoFisher Scientific) at the Hormel Institute, University of Minnesota. The movies were collected at a nominal magnification of 96,000×, corresponding to 0.89 Å per pixel, at a dose rate of 0.95 e^−^ per pixel per second with a defocus ranging from −1.0 to −2.4 µm. Each micrograph consists of 32 dose-framed fractions and was recorded with a total dose of 40 e^−^/Å^2^. The statistics of cryo-EM data collection are summarized in Supplementary Table 2.

### Image processing

Cryo-EM data were processed using cryoSPARC v3.3.1^33^, and the procedure is outlined in Supplementary Fig. 2 and Supplementary Fig. 3. In brief, dose-fractionated movies were subjected to Patch motion correction with MotionCor2^34^ and Patch CTF estimation with CTFFIND-4.1.13^35^. Particles were then automatically picked using Blob picker in cryoSPARC v3.3.1 with 384 pixels of the box size. Junk particles were removed through three rounds of 2D classifications. The 667,499 particles of the omicron spike and 348,980 particles of the prototypic spike from the good 2D classes were used for Ab-initio Reconstruction of six maps and four maps respectively. The initial models were set as the starting references for heterogeneous refinement (3D classification). The selected 3D classes were then subjected to further homogeneous, non-uniform and CTF refinements, generating the final maps. Particles in the good 3D class of the omicron spike were then imported into RELION-3.1^36^ using the csparc2star.py module^37^ and subjected to signal subtraction to keep only the standing-up RBD region, followed by masked 3D classification in RELION-3.1. Particles in the different classes from the masked 3D classification were further reverted to the original particles and subjected to non-uniform refinements in cryoSPARC v3.3.1. Resolutions of the maps were determined by gold-standard Fourier shell correlation (FSC) at 0.143 between the two half-maps. Local resolution variation was estimated from the two half-maps in cryoSPARC v3.3.1.

To perform further cleaning by iterative 3D classification and discarding of bad particles, we continued the next round of 3D classification for each of the three datasets. The results showed that the classes representing the bad particles have extremely small numbers compared with the good particles (Supplementary Fig. 4; 0.2–0.4% are bad particles), suggesting that the current particles are good for 3D reconstruction and that further iterative 3D classification to remove junk particles is unnecessary.

To verify the population statistics, we reprocessed data using template-based particle picking in cryoSPARC v3.3.1. Compared to blob particle picking, very similar results were obtained from template-based particle picking. The results are shown in Supplementary Fig. 5.

To rule out the potential existence of closed omicron spike and to support our results obtained from global and focused 3D classifications, we performed 3D variability for the omicron dataset (Supplementary Movies 1–4). We used the particles that generated the 3.0 Å resolution map to conduct 3D variability analysis in cryoSPARC v3.3.1 using 5 Å of filter resolution, 4 modes to solve, and all the other default parameters. The results were analyzed using 3D variability display in cryoSPARC v3.3.1 and were then displayed in individual movies in Chimera v1.16^38^. All these movies show the variability of the up RBD and the nearby NTD, but do not suggest the presence of the closed-form. The same procedure was also performed on the prototypic spike, revealing the presence of both prototypic open spike (Supplementary Movies 5–7) and prototypic closed spike (Supplementary Movies 8–10).

### Cryo-EM model building and refinement

Initial model building of the omicron open spike and the prototypic open spike was performed in Coot-0.8.9^39^ using PDB 7krr as a starting model, and the standing-up RBD was fitted into the density maps (both unsharpened and sharpened maps) as a rigid body. For the prototypic closed spike, PDB 7krq was used as a starting model. Several rounds of refinement in Phenix-1.16^40^ and manually building in Coot-0.8.9 were performed until the final reliable models were obtained. Model and map statistics are summarized in Supplementary Table 2. Figures were generated using UCSF Chimera X v0.93^41^ and PyMol v2.5.2^42^.

### Reporting summary

Further information on research design is available in the Nature Research Reporting Summary linked to this article.

## Supplementary information

Supplementary information

Description of Additional Supplementary Files

Supplementary Movie 1

Supplementary Movie 2

Supplementary Movie 3

Supplementary Movie 4

Supplementary Movie 5

Supplementary Movie 6

Supplementary Movie 7

Supplementary Movie 8

Supplementary Movie 9

Supplementary Movie 10

Reporting Summary

## Data Availability

The data that support this study are available from the corresponding authors upon reasonable request. The atomic models generated in this study have been deposited into the PDB with accession number 7TGW (omicron open spike), 7TGX (prototypic open spike), and 7TGY (prototypic closed spike). The corresponding cryo-EM density maps generated in this study have been deposited into the Electron Microscopy Data Bank with accession number EMD-25887 (omicron open spike), EMD-25888 (prototypic open spike), and EMD-25889 (prototypic closed spike).
