# Supplementary material for: Cryo-EM structure of a SARS-CoV-2 omicron spike protein ectodomain
Source: Nat Commun. 2022 Mar 3;13:1214. doi: 10.1038/s41467-022-28882-9 (PMC8894419; doi:10.1038/s41467-022-28882-9)
Supplement: Supplementary file 1 — Supplementary information [file 41467_2022_28882_MOESM1_ESM.pdf]

Supplementary information for

Cryo-EM structure of a SARS-CoV-2 omicron spike protein ectodomain

Gang Ye [*], Bin Liu [*,#], Fang Li [#]

* These authors contributed equally to this work.

# Correspondence:

Fang Li (lifang@umn.edu)
Bin Liu (liu00794@umn.edu)

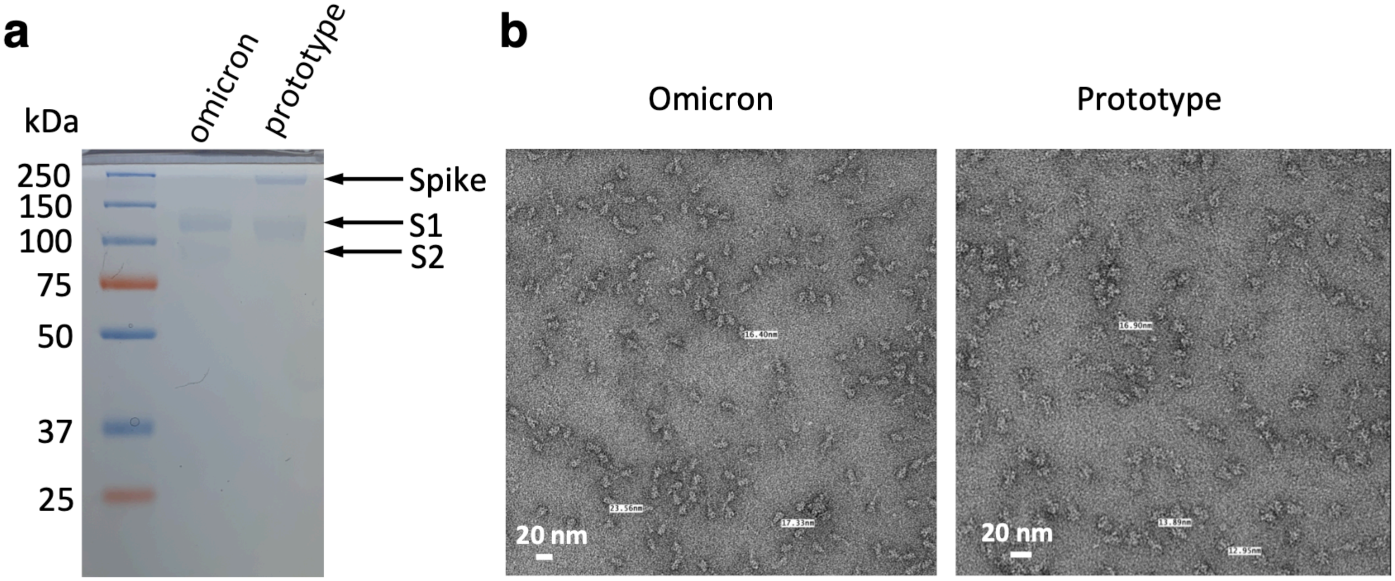

Supplementary Fig. 1: **Characterization of spike proteins.** (a) SDS-PAGE analyses (Coomasie Blue staining) of the recombinant SARS-CoV-2 omicron spike ectodomain and prototypic spike ectodomain. (b) The two protein samples were visualized in negative stain EM (left - omicron spike ectodomain; right - prototypic spike ectodomain). Scale bars are shown. The above experiments were repeated independently three times with similar results.

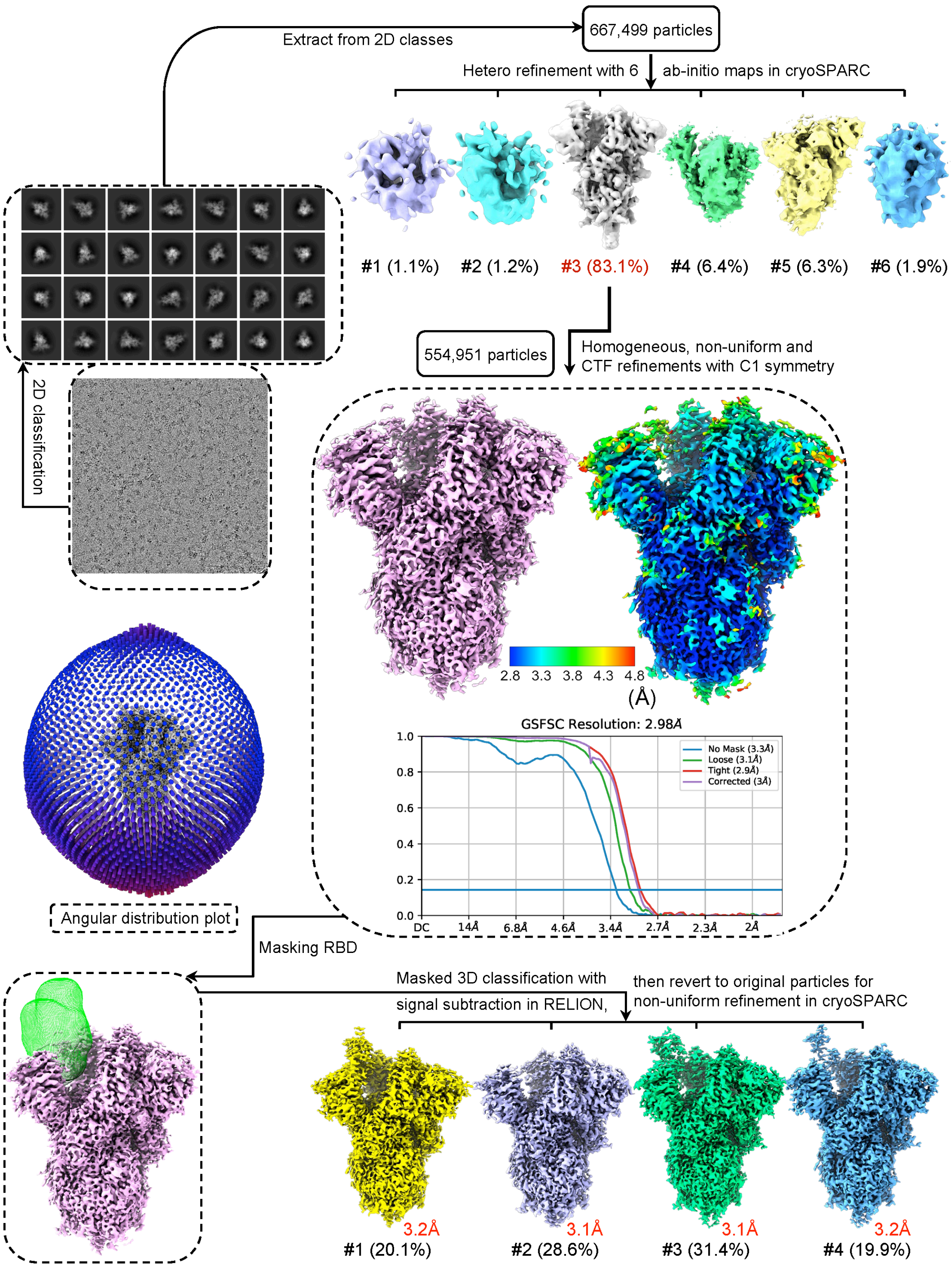

Supplementary Fig. 2: **Flow chart of cryo-EM image processing and map reconstruction for SARS-CoV-2 omicron spike after blob particle picking.** Representative raw cryo-EM image and 2D classes are presented. 3D refinement using all the particles in good 3D classes generated a 3.0 Å map. Further masked 3D classification generated four open conformations with mobile up-RBD. Angular distribution plot is displayed. The final maps, half-map FSC curves and accompanying local resolution illustrations are enclosed in the dashed black box.

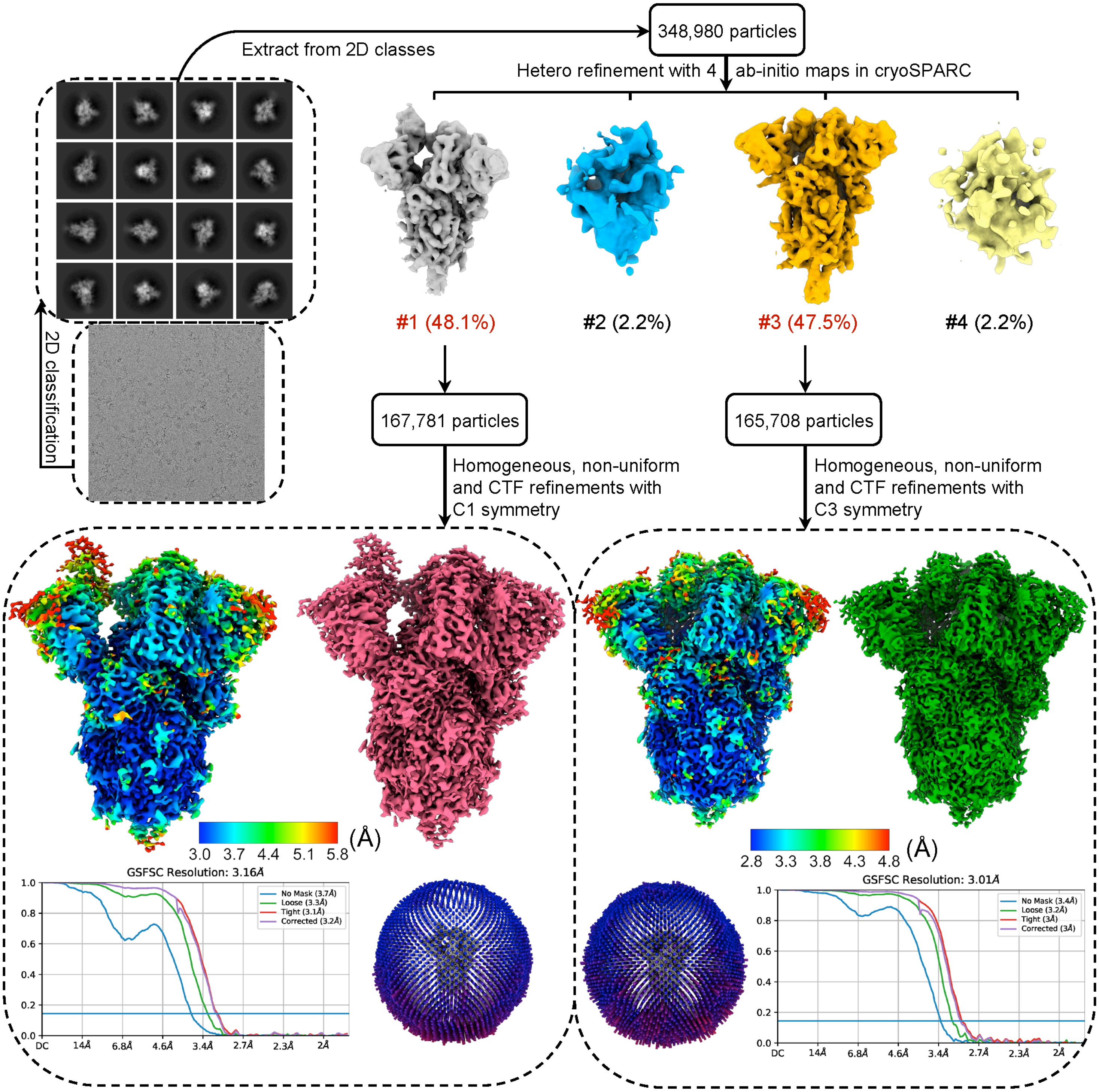

Supplementary Fig. 3: **Flow chart of cryo-EM image processing and map reconstruction for SARS-CoV-2 prototypic spike after blob particle picking.** Representative raw cryo-EM image and 2D classes are presented. 3D refinement using all the particles in good 3D classes generated a 3.2 Å and 3.0 Å map, respectively. The final maps, half-map FSC curves, angular distribution plot and accompanying local resolution illustrations are enclosed in the dashed black box.

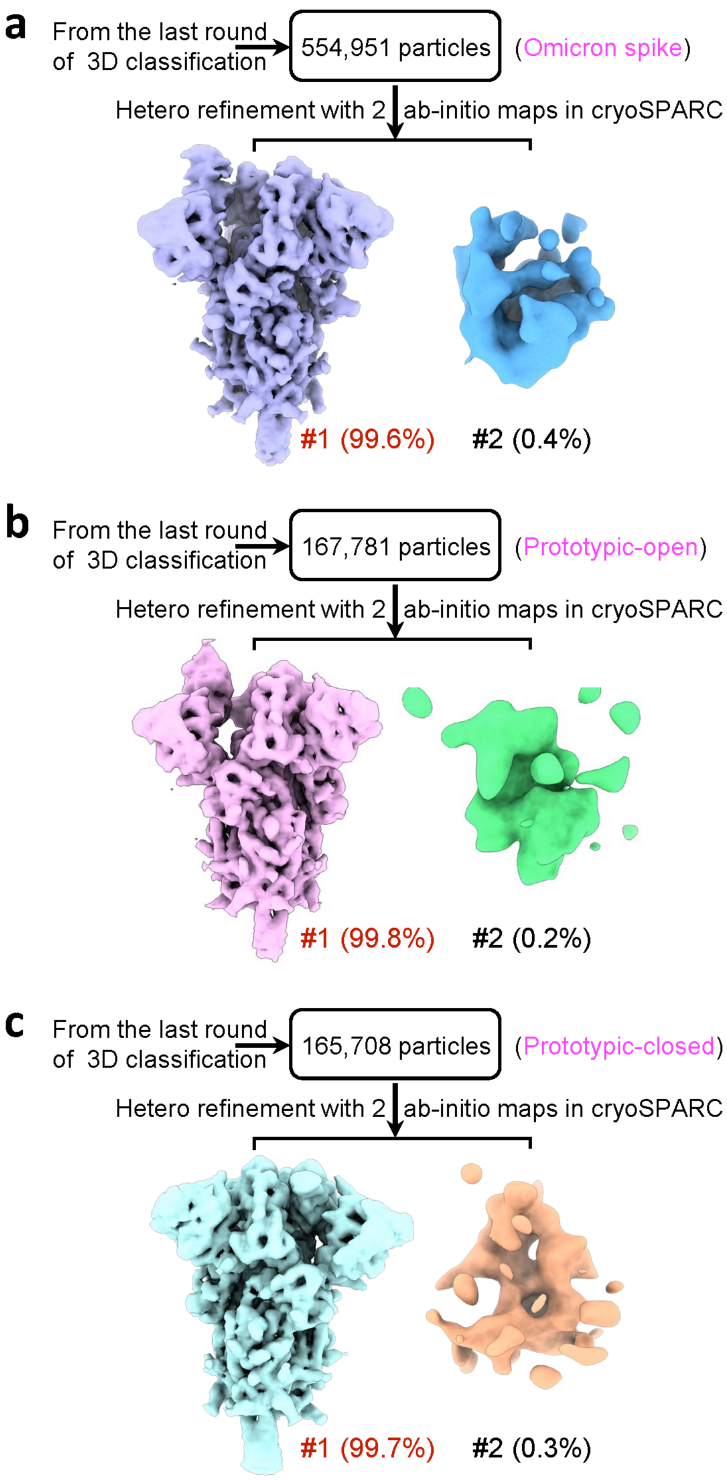

Supplementary Fig. 4: **A further round 3D classification for each of the three datasets:** (a) omicron spike; (b) prototypic open spike; (c) prototypic closed spike. The results showed that the classes representing the bad particles have extremely small numbers (0.2%-0.4%) compared with the good particles, suggesting the current particles are good for 3D reconstruction.

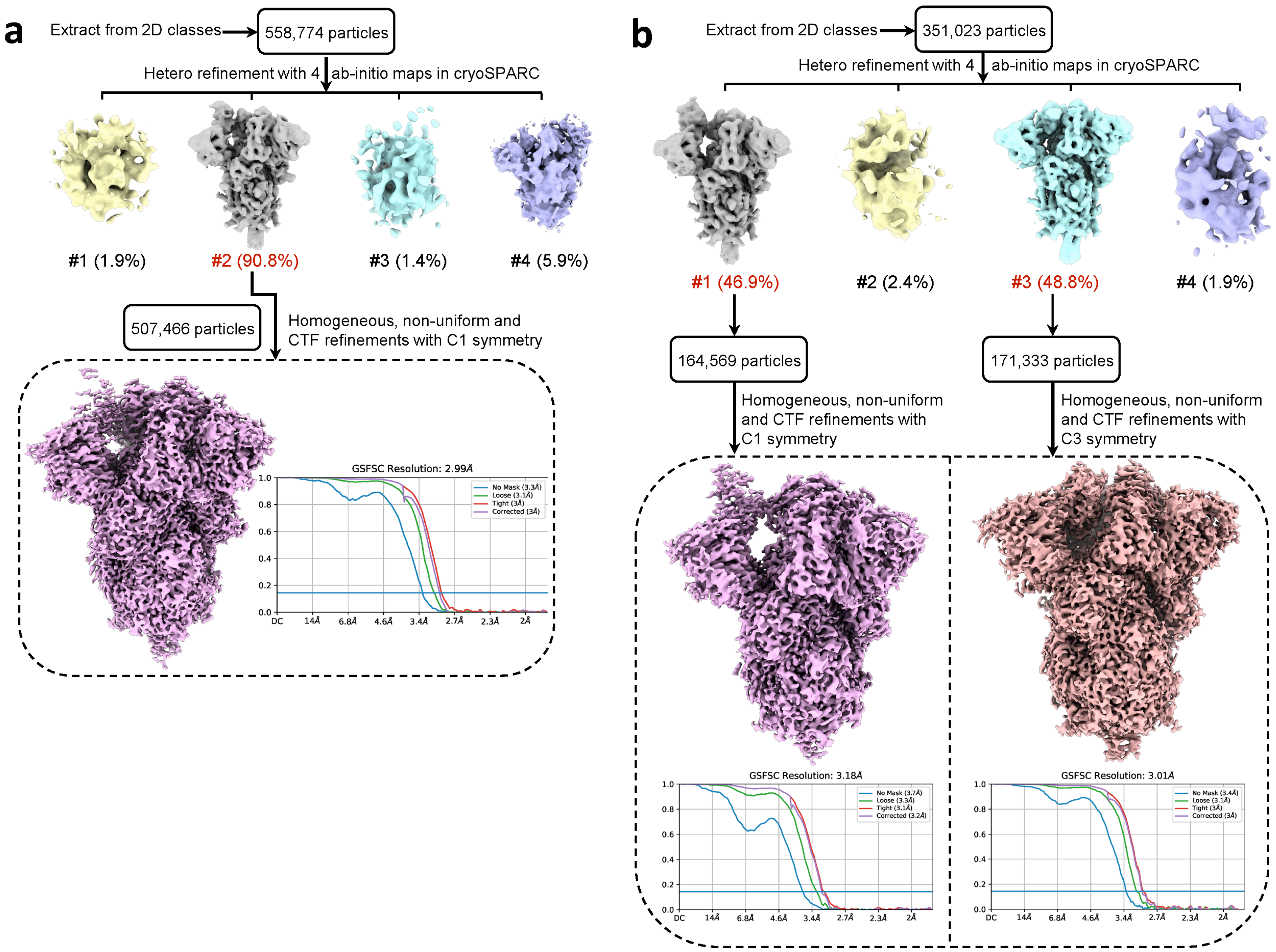

Supplementary Fig. 5: **Flow charts of cryo-EM image processing and map reconstructions for SARS-CoV-2 spike after template-based particle picking:** (a) omicron spike; (b) prototypic spike. 3D refinement using all the particles in good 3D classes generated a 3.0 Å map for the omicron spike and a 3.2 or 3.0 Å map for the prototypic open or closed spike, respectively. The final maps and half-map FSC curves are enclosed in the dashed black box.

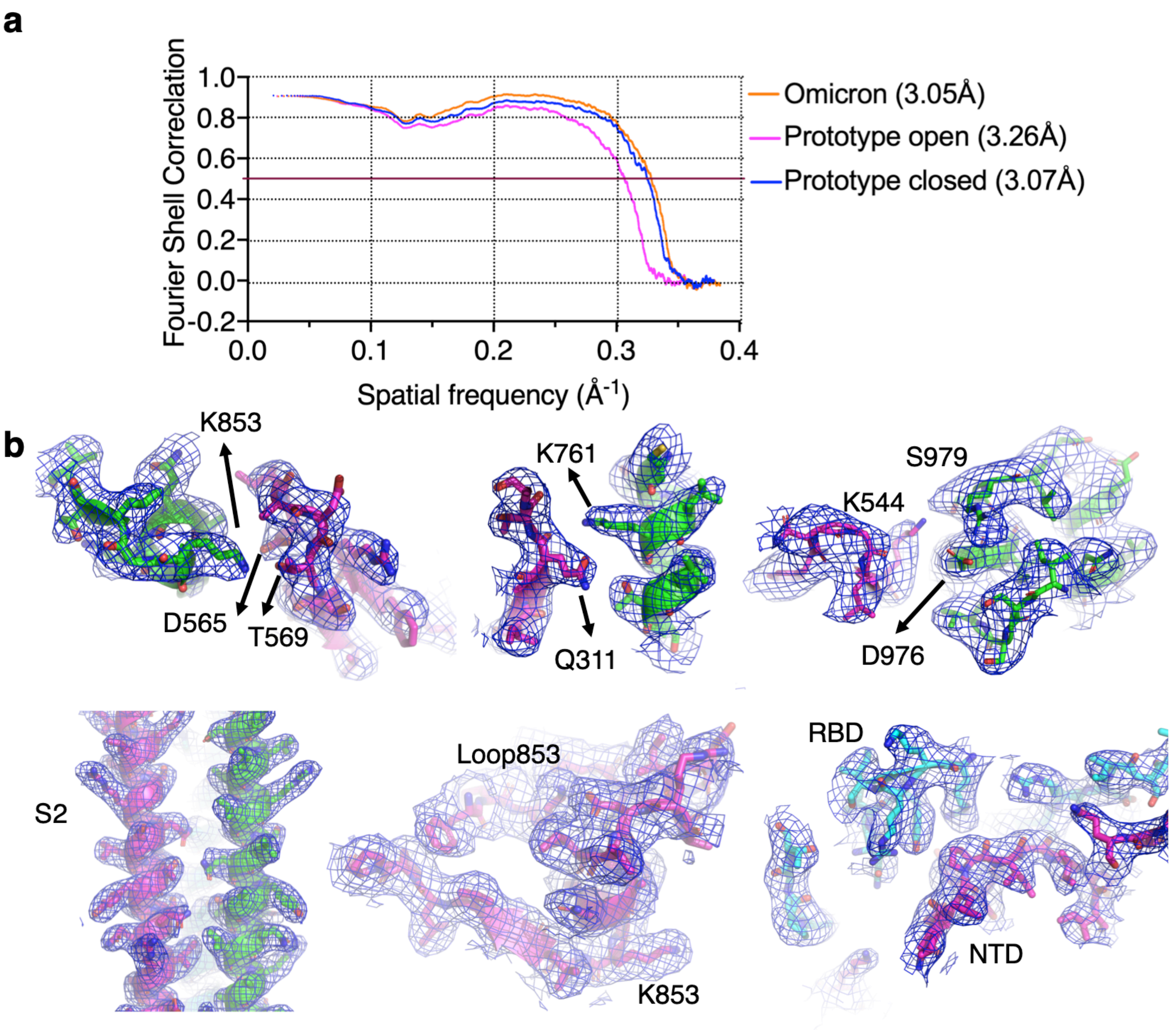

Supplementary Fig. 6: **Cryo-EM validations.** (a) Model-to-map FSC curves for omicron open spike, prototypic open spike and prototypic closed spike. (b) Cryo-EM densities of selected regions in the omicron open spike. Top panels: densities of the regions displayed in Fig. 3b, Fig. 3c and Fig. 3d, respectively. Bottom panels: densities of the S2 central helices, loop 853 and RBD/NTD interface. Residues are shown as sticks and colored by subunits and elements.

Supplementary Table 1: **Summary of the RBD conformations of the SARS-CoV-2 spike.**

<table>
<tr><th>Spike</th><th>Reference</th><th>Cleaved</th><th>Buffer</th><th>Closed</th><th>1-RBD up</th><th>2-RBD up</th><th>3-RBD up</th><th>Inter-mediate RBD</th></tr>
<tr><td>Omicron</td><td>This study</td><td>Yes</td><td>20mM Tris pH7.4, 200mM NaCl, 8mM CHAPSO</td><td></td><td>~100%</td><td></td><td></td><td></td></tr>
<tr><td rowspan="2">Wuhan</td><td rowspan="2">1</td><td>Yes</td><td rowspan="2">20mM Tris pH8.0, 150mM NaCl, 0.1% OG</td><td>34%</td><td>27%</td><td></td><td></td><td>39%</td></tr>
<tr><td>No</td><td>83%</td><td>17%</td><td></td><td></td><td></td></tr>
<tr><td rowspan="5">Prototype (Wuhan + D614G)</td><td>This study</td><td>Yes</td><td>Same as omicron</td><td>49.7%</td><td>50.3%</td><td></td><td></td><td></td></tr>
<tr><td>2</td><td>No</td><td>10nm colloidal gold in PBS</td><td>31%</td><td>55%</td><td>14%</td><td></td><td></td></tr>
<tr><td>3</td><td>No</td><td>25mM NaHEPES pH7.4, 150mM NaCl</td><td>5%</td><td>36%</td><td>39%</td><td>20%</td><td></td></tr>
<tr><td rowspan="2">4</td><td>No</td><td rowspan="2">2mM Tris pH8.0, 200mM NaCl, 0.02% NaN_3</td><td>56%</td><td>44%</td><td></td><td></td><td></td></tr>
<tr><td>Yes</td><td>69%</td><td>31%</td><td></td><td></td><td></td></tr>
<tr><td>Gamma</td><td>5</td><td>No</td><td>2mM Tris pH8.0, 200mM NaCl, 0.02% NaN3, 0.5% glycerol</td><td>15%</td><td colspan="2">85%</td><td></td><td></td></tr>
<tr><td>Alpha</td><td rowspan="2">6</td><td rowspan="2">Yes</td><td rowspan="5">25mM Tris pH7.5, 150mM NaCl, 0.02%DDM</td><td>16%</td><td>81%</td><td>3%</td><td></td><td></td></tr>
<tr><td>Beta</td><td>24%</td><td>76%</td><td></td><td></td><td></td></tr>
<tr><td>Delta</td><td rowspan="3">7</td><td rowspan="3">Yes</td><td>31%</td><td>69%</td><td></td><td></td><td></td></tr>
<tr><td>Kappa</td><td>54%</td><td>46%</td><td></td><td></td><td></td></tr>
<tr><td>Gamma</td><td></td><td>100%</td><td></td><td></td><td></td></tr>
</table>

1 Wrobel, A. G. *et al.* SARS-CoV-2 and bat RaTG13 spike glycoprotein structures inform on virus evolution and furin-cleavage effects. *Nat Struct Mol Biol* **27**, 763-767, doi:10.1038/s41594-020-0468-7 (2020)

2 Ke, Z. *et al.* Structures and distributions of SARS-CoV-2 spike proteins on intact virions. *Nature* **588**, 498-502, doi:10.1038/s41586-020-2665-2 (2020).

3 Yurkovetskiy, L. *et al.* Structural and Functional Analysis of the D614G SARS-CoV-2 Spike Protein Variant. *Cell* **183**, 739-751 e738, doi:10.1016/j.cell.2020.09.032 (2020).

4 Gobeil, S. M. *et al.* D614G Mutation Alters SARS-CoV-2 Spike Conformation and Enhances Protease Cleavage at the S1/S2 Junction. *Cell Rep* **34**, 108630, doi:10.1016/j.celrep.2020.108630 (2021).

5 Gobeil, S. M. *et al.* Effect of natural mutations of SARS-CoV-2 on spike structure, conformation, and antigenicity. *Science* **373**, doi:10.1126/science.abi6226 (2021).

6 Cai, Y. *et al.* Structural basis for enhanced infectivity and immune evasion of SARS-CoV-2 variants. *Science* **373**, 642-648, doi:10.1126/science.abi9745 (2021).

7 Zhang, J. *et al.* Membrane fusion and immune evasion by the spike protein of SARS-CoV-2 Delta variant. *Science* **374**, 1353-1360, doi:10.1126/science.abl9463 (2021).

Supplementary Table 2: **Cryo-EM data collection, refinement and validation statistics**

Spike	Omicron (EMDB-25887) (PDB 7TGW) Open	Prototype (EMDB-25888) (PDB 7TGX) Open	Prototype (EMDB-25889) (PDB 7TGY) Closed
**Data collection and processing**			
Magnification	96,000	96,000	96,000
Voltage (kV)	300	300	300
Electron exposure (e–/Å^2)	40.00	40.00	40.00
Defocus range (μm)	1.0–2.4	1.0–2.4	1.0–2.4
Pixel size (Å)	0.89	0.89	0.89
Symmetry imposed	C1	C1	C3
Initial particle images (no.)	667,499	348,980	348,980
Final particle images (no.)	554,951	167,781	165,708
Map resolution (Å)	3.0	3.2	3.0
FSC threshold	0.143	0.143	0.143
Map resolution range (Å)	2.8–4.8	3.0–5.8	2.8–4.8
**Refinement**			
Initial model used (PDB code)	7krr	7krr	7krq
Model resolution (Å)	3.05	3.26	3.07
FSC threshold	0.5	0.5	0.5
Model resolution range (Å)	40.4-2.8	41.7-2.9	47.9-2.8
Map sharpening *B* factor (Å^2)	-132.8	-118.4	-136.3
Model composition			
Non-hydrogen atoms	26617	25778	25999
Protein residues	3307	3244	3254
Ligands	45	32	42
B factors (Å^2)			
Protein	117.76	127.30	118.45
Nucleotide			
Ligand	104.17	110.47	115.82
R.m.s. deviations			
Bond lengths (Å)	0.007	0.005	0.010
Bond angles (°)	0.828	0.626	0.815
Validation			
MolProbity score	1.99	1.84	1.97
Clashscore	10.05	7.37	9.2
Poor rotamers (%)	0.17	0.18	0.18
Ramachandran plot			
Favored (%)	92.63	93.39	92.14
Allowed (%)	7.34	6.58	7.83
Disallowed (%)	0.03	0.03	0.03

Supplementary Table 3: **Summary of buried inter-domain and inter-subunit surfaces**

Interface ($Å^2$)	RBD/NTD interface		S1/S2 interface		
	RBD (subunit B)/ NTD (subunit C)	RBD (subunit C)/ NTD (subunit A)	S1 (subunit A)/ S2 (subunit B)	S1 (subunit B)/ S2 (subunit C)	S1 (subunit C)/ S2 (subunit A)
Omicron spike (open)	570.5	504.1	1196.8	1550.9	1610.7
Prototypic spike (open)	122.4	142	880.2	1100.3	1170.5

All the buried surfaces are calculated by using the online tool, PDBePISA v1.52 (https://www.ebi.ac.uk/pdbe/pisa/). Subunit A contains the up-RBD, and subunits B and C both contain a down-RBD.
