# Supplementary material for: Cryo-EM structure of a SARS-CoV-2 omicron spike protein ectodomain
Source: Nat Commun. 2022 Mar 3;13:1214. doi: 10.1038/s41467-022-28882-9 (PMC8894419; doi:10.1038/s41467-022-28882-9)
Supplement: Supplementary file 2 — Description of Additional Supplementary Files [file 41467_2022_28882_MOESM2_ESM.pdf]

Description of Additional Supplementary Files

File Name: Supplementary Movie 1
Description: 3D variability analysis of SARS-CoV-2 omicron spike ectodomain (side view). The first of the four different orthogonal principle modes was generated in 3D variability analysis on the final good particles for 3D refinement and is displayed in blue.

File Name: Supplementary Movie 2
Description: 3D variability analysis of SARS-CoV-2 omicron spike ectodomain (side view). The second of the four different orthogonal principle modes was generated in 3D variability analysis on the final good particles for 3D refinement and is displayed in purple.

File Name: Supplementary Movie 3
Description: 3D variability analysis of SARS-CoV-2 omicron spike ectodomain (side view). The third of the four different orthogonal principle modes was generated in 3D variability analysis on the final good particles for 3D refinement and is displayed in yellow.

File Name: Supplementary Movie 4
Description: 3D variability analysis of SARS-CoV-2 omicron spike ectodomain (side view). The fourth of the four different orthogonal principle modes was generated in 3D variability analysis on the final good particles for 3D refinement and is displayed in green.

File Name: Supplementary Movie 5
Description: 3D variability analysis of SARS-CoV-2 prototypic spike ectodomain in the open conformation (side view). The first of the three different orthogonal principle modes was generated in 3D variability analysis on the final good particles for 3D refinement and is displayed in blue.

File Name: Supplementary Movie 6
Description: 3D variability analysis of SARS-CoV-2 prototypic spike ectodomain in the open conformation (side view). The second of the three different orthogonal principle modes was generated in 3D variability analysis on the final good particles for 3D refinement and is displayed in purple.

File Name: Supplementary Movie 7
Description: 3D variability analysis of SARS-CoV-2 prototypic spike ectodomain in the open conformation (side view). The third of the three different orthogonal principle modes was generated in 3D variability analysis on the final good particles for 3D refinement and is displayed in yellow.

File Name: Supplementary Movie 8
Description: 3D variability analysis of SARS-CoV-2 prototypic spike ectodomain in the closed conformation (top view). The first of the three different orthogonal principle

modes was generated in 3D variability analysis on the final good particles for 3D refinement and is displayed in blue.

File Name: Supplementary Movie 9
Description: 3D variability analysis of SARS-CoV-2 prototypic spike ectodomain in the closed conformation (top view). The second of the three different orthogonal principle modes was generated in 3D variability analysis on the final good particles for 3D refinement and is displayed in purple.

File Name: Supplementary Movie 10
Description: 3D variability analysis of SARS-CoV-2 prototypic spike ectodomain in the closed conformation (top view). The third of the three different orthogonal principle modes was generated in 3D variability analysis on the final good particles for 3D refinement and is displayed in yellow.
