# Supplementary material for: Cryo-EM structure of a SARS-CoV-2 omicron spike protein ectodomain
Source: Nat Commun. 2022 Mar 3;13:1214. doi: 10.1038/s41467-022-28882-9 (PMC8894419; doi:10.1038/s41467-022-28882-9)
Supplement: Supplementary file 13 — Reporting Summary [file 41467_2022_28882_MOESM13_ESM.pdf]

nature portfolio

Corresponding author(s): Bin Liu, Fang Li

Last updated by author(s): Feb 16, 2022

Reporting Summary

Nature Portfolio wishes to improve the reproducibility of the work that we publish. This form provides structure for consistency and transparency in reporting. For further information on Nature Portfolio policies, see our Editorial Policies and the Editorial Policy Checklist.

Statistics

For all statistical analyses, confirm that the following items are present in the figure legend, table legend, main text, or Methods section.

n/a	Confirmed	
☒	☐	The exact sample size (n) for each experimental group/condition, given as a discrete number and unit of measurement
☒	☐	A statement on whether measurements were taken from distinct samples or whether the same sample was measured repeatedly
☒	☐	The statistical test(s) used AND whether they are one- or two-sided<br>*Only common tests should be described solely by name; describe more complex techniques in the Methods section.*
☒	☐	A description of all covariates tested
☒	☐	A description of any assumptions or corrections, such as tests of normality and adjustment for multiple comparisons
☒	☐	A full description of the statistical parameters including central tendency (e.g. means) or other basic estimates (e.g. regression coefficient) AND variation (e.g. standard deviation) or associated estimates of uncertainty (e.g. confidence intervals)
☒	☐	For null hypothesis testing, the test statistic (e.g. F, t, r) with confidence intervals, effect sizes, degrees of freedom and P value noted<br>*Give P values as exact values whenever suitable.*
☒	☐	For Bayesian analysis, information on the choice of priors and Markov chain Monte Carlo settings
☒	☐	For hierarchical and complex designs, identification of the appropriate level for tests and full reporting of outcomes
☒	☐	Estimates of effect sizes (e.g. Cohen's d, Pearson's r), indicating how they were calculated

Our web collection on statistics for biologists contains articles on many of the points above.

Software and code

Policy information about availability of computer code

Data collection	EPU version 2.5
Data analysis	RELION-3.1; MotionCor2; CTFFIND-4.1.13; cryoSPARC v3.3.1; Blob Picker (a program in cryoSPARC v3.3.1); Template-based Picker (a program in cryoSPARC v3.3.1); 3D variability (a program in cryoSPARC v3.3.1); UCSF pyem v0.5; Phenix-1.16; Coot-0.8.9; UCSF Chimera v1.16; UCSF ChimeraX v0.93; PyMol v2.5.2; PDBePISA web server v1.52

For manuscripts utilizing custom algorithms or software that are central to the research but not yet described in published literature, software must be made available to editors and reviewers. We strongly encourage code deposition in a community repository (e.g. GitHub). See the Nature Portfolio guidelines for submitting code & software for further information.

Data

Policy information about availability of data

All manuscripts must include a data availability statement. This statement should provide the following information, where applicable:

- Accession codes, unique identifiers, or web links for publicly available datasets
- A description of any restrictions on data availability
- For clinical datasets or third party data, please ensure that the statement adheres to our policy

The atomic models generated in this study have been deposited into the PDB with accession number 7TGW (https://www.rcsb.org/structure/7TGW) (omicron open spike), 7TGX (https://www.rcsb.org/structure/7TGX) (prototypic open spike) and 7TGY (https://www.rcsb.org/structure/7TGY) (prototypic closed spike).
The cryo-EM density maps generated in this study have been deposited into the Electron Microscopy Data Bank with accession number EMD-25887 (https://www.ebi.ac.uk/pdbe/entry/emdb/EMD-25887) (omicron open spike), EMD-25888 (https://www.ebi.ac.uk/pdbe/entry/emdb/EMD-25888) (prototypic open spike) and EMD-25889 (https://www.ebi.ac.uk/pdbe/entry/emdb/EMD-25889) (prototypic closed spike).

Field-specific reporting

Please select the one below that is the best fit for your research. If you are not sure, read the appropriate sections before making your selection.

[x] Life sciences   [ ] Behavioural & social sciences   [ ] Ecological, evolutionary & environmental sciences

For a reference copy of the document with all sections, see nature.com/documents/nr-reporting-summary-flat.pdf

Life sciences study design

All studies must disclose on these points even when the disclosure is negative.

Sample size	No sample size calculations were performed. For cryo-EM samples, six grids of either the omicron spike or the prototypic spike were pre-screened to identify the optimal grid for data collection. Sufficient cryo-EM raw movies were collected until cryo-EM maps with adequate resolutions for model building can be achieved.
Data exclusions	No data were excluded from analyses.
Replication	Cryo-EM single particle analysis inherently relied on averaging over a large number of independent observations. During the processing pipeline, replicate reconstructions were calculated over 3 times during the related refinement procedures, yielding the same results at better and better resolutions.
Randomization	Randomization was not relevant to our study because our study did not involve the allocation of samples/organisms/participants into experimental groups.
Blinding	Investigators were not blinded to group allocation because group allocation was not involved in our study. Investigators were not blinded during data collection because the data being collected were quantitative in nature (gels or numbers of colonies on a plate) and were not prone to subjective interpretation.

Reporting for specific materials, systems and methods

We require information from authors about some types of materials, experimental systems and methods used in many studies. Here, indicate whether each material, system or method listed is relevant to your study. If you are not sure if a list item applies to your research, read the appropriate section before selecting a response.

Materials & experimental systems

n/a	Involved in the study	
[x]	[ ]	Antibodies
[ ]	[x]	Eukaryotic cell lines
[x]	[ ]	Palaeontology and archaeology
[x]	[ ]	Animals and other organisms
[x]	[ ]	Human research participants
[x]	[ ]	Clinical data
[x]	[ ]	Dual use research of concern

Methods

n/a	Involved in the study	
[x]	[ ]	ChIP-seq
[x]	[ ]	Flow cytometry
[x]	[ ]	MRI-based neuroimaging

Eukaryotic cell lines

Policy information about cell lines

Cell line source(s)	FreeStyle™ 293-F Cells (ThermoFisher)
Authentication	The cell line was purchased from ThermoFisher and was not authenticated
Mycoplasma contamination	The cell line was used for protein expression and were not tested for mycoplasma contamination
Commonly misidentified lines (See ICLAC register)	No commonly misidentified cell lines were used
